# 2327: Prescription opioid dependence in Western New York: Using data analytics to find an answer to the opioid epidemic

**DOI:** 10.1017/cts.2017.69

**Published:** 2018-05-10

**Authors:** Shyamashree Sinha, Gale Burstein, Kenneth E. Leonard, Timothy Murphy, Peter Elkin

**Affiliations:** University at Buffalo, State University of New York, Buffalo, NY, USA

## Abstract

OBJECTIVES/SPECIFIC AIMS: Dependence and abuse of prescription opioid pain medication has substantially increased over the last decade. The consistent rise in opioid dependence contributes to the rising prescription drug overdose deaths over the last decade. The study of the distribution and determinants of opioid dependence among patients who are treated with chronic pain medications prescribed by their healthcare providers would aid in answering some key questions about potential abuse and overdose on opioids. The descriptive epidemiology of opioid dependence would help in identifying the vulnerable age group, race, ethnicity, and type of opioid pain medications that more commonly result in dependence. METHODS/STUDY POPULATION: We implemented an Observational Medical Outcomes Partnership/Observational Health Data Sciences and Informatics (OMOP/OHDSI) database, to hold structured EHR data from our Allscripts patient records. We also created a high-throughput phenotyping, natural language processing system that can parse 7,000,000 clinical notes in 1.5 hours. This runs as a web service and provides a modular component based NLP system. After the full semantic parse, we match the content against any number of ontologies. For each match we tag it as either a positive, negative, or uncertain assertion. We then perform automated compositional expressions. The codes are stored in a Berkley database (BDB) NOSQL database and the compositional expressions are stored in Neo4J (a graph database) and Graph DB (a triple store). This flexibility allows rapid retrieval of complex questions in real time. The High-Throughput Phenotyping (HTP) Natural Language Processing (NLP) Subsystem (HTP-NLP) is software that produces, given biomedical text, semantic annotations of the text. The semantic annotations identify conceptual entities—their attributes, the relations they have with other entities and the events they participate in, as expressed in the input text. The conceptual entities, relations, attributes, and events identified are specified by various knowledge representations (KRs) as documented in Coding Sources. Examples of coding sources are medical terminologies [eg, SNOMED CT, RxNorm, LOINC and open biomedical ontologies (OBO) foundry ontologies, eg, gene ontology (GO), functional model of anatomy, OBI, and others]. The annotation results may be displayed or output in formats suitable for further processing. Entity identified is assigned a truth value from 0 to 1. Values from the text are assigned to entities from ontologies such as SNOMED CT. The retrospective analysis of EHR data from local clinic patients was performed using queries on the problem list, demographic data, and medication list of all the patients in the database. The OMOP/OHDSI database was collected from Allscripts EHRs from 2010 to 2015. This common data model helps in the systematic analysis of disparate observational databases of clinic records from the primary care and family medicine clinics in Western New York region. The database contained 212,343 patient records that were parsed and deidentified. Specific research IDs were assigned to each of the patient records and stored in a secure firewall device for data analytics. The entire 212,343 records were queried for opioid dependence from the ICD-9 and 10 diagnostic codes and SNOMED CT codes mapped to both the clinical notes and the problem list for each patient based on the mapped ICD and SNOMED CT codes. In total, 1356 patients were identified as to having opioid dependence. The records were stratified into 7 age groups from age 18 to 28 and ending with age 79–89 years. RESULTS/ANTICIPATED RESULTS: Of the 212,343 patients in the database 1356 patients revealed opioid dependence on the problem list, ICD9-10 codes and prescription opioid pain medication with or without Buprenorphine and Naloxone (Suboxone) in the medication list. The prevalence of opioid dependence in the clinic population was 0.64% (95% CI: 0.61%–0.67%) over a 5-year period. The 7,000,000 patient records generated 750,000,000 SNOMED CT codes (on average 107 codes per record). The highest numbers of opioid dependence were seen in the 29 to 38 years’ age group. That comprised 39.38% (95% CI: 36.78%–41.98%) of the total opioid dependent population but accounted for only 2.03% of whole clinic population in this age group (95% CI: 1.86% to 2.2%). The subjects were then stratified by race and ethnicity. There were 1005 patients with opioid dependence, in the non-Hispanic population (total number 108,402). Among the White non-Hispanic or Latino population with opioid dependence, 41.33% (95% CI: 38.27%–44.39%) were 29–38 years old. The next common age group among the White Non-Hispanic opioid dependent subjects was 19–28 years, comprising of 22.48% (95% CI: 19.88%–25.08%) of the total number of White non-Hispanic or Latino opioid dependent population. Among the total clinic population Hispanics comprise 51.24%, but they comprise only 2.58% (95% CI: 1.74%–3.42%) of the total opioid dependent population. The non-Hispanic population comprise 51.05% of total clinic population while the percent of people who are opioid dependent is 83.26% (95% CI: 83.04%–83.48%) of the total 1356 opioid dependent population. DISCUSSION/SIGNIFICANCE OF IMPACT: The trends of opioid dependence among the clinic population in the study indicate that the prevalence is more in a certain section of the population. The predominance is among the non-Hispanic White population in the 19–38 years of age. The prevalence in younger age implies that the complications related to opioid dependence would be there for a longer duration of time. The prevalence of dependence in this clinic population would be rising if this trend continues. Interventions at curbing prescription opioid dependence is necessary for the vulnerable population. The findings suggest that a broad based approach is necessary to address this problem. The distribution of opioid dependence in this patient population indicate the need for special attention to these specific age group and race ethnicities. The young age of many of the addicted patients demonstrate the risks of legitimate opioid prescriptions in leading this age group towards addiction and implies the need for routine screening for substance abuse. The evidence of complications of opioid overdose among long-term opioid users and risk of abuse with other agents including illicit agents makes the need for an approach that uses real-time interventions in addition to effect long-term improvement in addiction rates. A potentially cost-effective approach to implement monitoring programs and clinical decision support tools would be to develop inter operable linkage from the EHRs to the state Department of Healths’ prescription monitoring programs.

